# Transformation of struvite from wastewater to a hydrogen fuel storage compound ammonia borane

**DOI:** 10.3389/fchem.2023.1269845

**Published:** 2023-11-07

**Authors:** Nin Dingra, Michael Witty, Marie Celis, Narendra Boppana, Theppawut Ayudhya

**Affiliations:** ^1^ Department of Chemistry, University of Texas Permian Basin, Odessa, TX, United States; ^2^ School of Pure and Applied Sciences, Florida SouthWestern State College, Fort Myers, FL, United States; ^3^ Department of Chemical Engineering, University of Texas Permian Basin, Odessa, TX, United States

**Keywords:** ammonia borane, hydrogen storage, struvite, sustainable energy, urine, wastewater

## Abstract

Ammonia borane (NH_3_BH_3_) is a carrier of hydrogen gas that is known as a carbon-free renewable energy source. A high hydrogen content of ammonia borane and its stability in air at ambient temperatures make it a valuable molecule for its potential use as a hydrogen storage compound. In this study, we investigate a new approach for synthesizing ammonia borane using wastewater-derived ammonia source. Wastewater recycling has always been a global interest towards sustainability. In addition to reclaiming the water, recycling nutrients in wastewater is a topic of interest. Nutrients such as nitrogen, magnesium, and phosphorous are readily recovered from wastewater as struvite (NH_4_MgPO_4_·6H_2_O). This new process involves converting urine into struvite, and then reacting struvite with alkali borohydrides to produce a high-purity ammonia borane. The use of mild reaction conditions without extensive purification process, together with high purity ammonia borane product make this process a desirable course of action for recycling the nitrogen waste. In the course of moving towards a sustainable environment, the energy and wastewater industries will benefit from this combined process of nitrogen removal from wastewater to generate a renewable carbon-free energy molecule.

## 1 Introduction

Nutrient recovery from wastewater, specifically human excreta, has been a major focus of ecological sanitation, an approach that allows safe recycling of nutrients rather than discarding them into the freshwater bodies (Esrey, 2001; Simha and Ganesapillai, 2007). The chemical composition of human excreta contains various important nutrients including nitrogen (N), potassium (K), and phosphorous (P). Although human urine accounts for less than 1% of total wastewater volume, more than 80% of total-N, more than 50% of total-K and total-P are found in urine (Kirchmann and Pettersson, 1994). Therefore, source-separation systems are optimal for recovering N and P in maximum amounts compared to the conventional wastewater treatment systems (Malila et al., 2019). These nutrients are often recovered in the form of struvite, a white crystalline substance consisting of equal molar amounts of ammonium, magnesium, and phosphate with the formula of NH_4_MgPO_4_·6H_2_O. In solutions that contain magnesium, ammonium, and phosphate, struvite forms crystals under the optimal pH values (Doyle and Parsons, 2002). Struvite crystals have several possible shapes including a distinctive form with triangular faces known as the Coffin Lid form which we will refer to later (Witty, 2016b). Struvite forms spontaneously in wastewater and is often found in the pipes of sewerage treatment plants causing blockages and reducing water flow. Due to this, many have developed methods for effective, frequent, and fast removal of struvite. A pilot-scale and long-term treatment of source separated urine to recover phosphorus and nitrogen as struvite has also been reported (Zamora et al., 2017) making struvite readily accessible to be used as nitrogen and phosphorus source in the agricultural industry as fertilizer. Although many excellent technologies exist for harvesting these nutrients (Maroušek et al., 2020) there is still great interest in struvite technology which is growing exponentially.

The recycled nutrients from wastewater can also be directed towards other purposes such as making energy storage molecules in the energy industry. An increase in energy demand and consumption, along with a desire to reduce fossil fuel utilization, creates a demand for finding ways to transition to more carbon free and renewable energy sources (Veziroglu, 2007; Maaß and Möckel, 2019). The new sources should be eco-friendly and must also be sustainable, abundant, and have high energy density. Although wind and solar energy are abundant, the main obstacle for capturing energy from these sources is energy storage and intermittent availability. Hydrogen fuel appears to fit the desired qualities needed for a clean energy solution (Acar and Dincer, 2020). However, the challenges for storage and transport of gaseous H_2_ involving high pressure (up to 80 MPa) and liquefaction (−253°C) remain major obstacles in the use of hydrogen gas as fuel (Durbin and Malardier-Jugroot, 2013). These shortcomings led researchers to discover safer and more effective ways of storing hydrogen fuel. A solution to resolve this problem is to use solid hydrides or solid absorbent materials for hydrogen storage (Stephens et al., 2007).

The study of complex solid hydrides, such as sodium borohydride (NaBH_4_), lithium borohydride (LiBH_4_), sodium aluminum hydride (NaAlH_4_), magnesium borohydride [Mg(BH_4_)_2_], and lastly ammonia borane (NH_3_BH_3_) as potential sources of hydrogen gas and the development of ways to release it from the solids is a rapidly developing field of research (Puszkiel et al., 2017; Akbayrak and Özkar, 2018). Ammonia borane (AB), NH_3_BH_3_, is of particular interest since it has a very high hydrogen content (19.6 wt%) and is highly stable in the air at ambient temperatures (Komova et al., 2013). The release of hydrogen from AB starts immediately upon its melting, around 100°C, leaving polymeric structures that can release more hydrogen at 130°C (Hamilton et al., 2009). Hydrogen liberation from AB is significantly improved by using transition metal catalyst such as TiO_2_ with water adsorbed, which allowed for the hydrogen release at 80°C (Komova et al., 2013; Zhang et al., 2017). Overall, ammonia borane represents an optimal hydrogen source in terms of the stability of its crystal form, transport, and hydrogen release process. Despite its potential in the field of hydrogen energy, the availability of highly pure ammonia borane still remains a challenge.

Various methods for the synthesis of ammonia borane have been reported in the literature (Li et al., 2014; Hirscher et al., 2020). Early reports on preparation of AB accounted the decomposition of the diammoniate of diborane in ether or polyether solutions (Shore et al., 1958; Shore and Parry, 1958). Another approach for AB synthesis uses displacement reactions where borane is transferred from a weak base such as THF or DMS to a strong base, NH_3_ (Beres et al., 1971; Jaska et al., 2003). The yield and purity of AB obtained from these methods are less attractive due to the formation of unstable intermediates such as ammonia diborane, diammonia diborane, and other ionic by-products (Chen et al., 2013). The most prevalent method for preparation of AB is metathesis reaction that use borohydrides and ammonium salts (Ramachandran and Gagare, 2007; Heldebrant et al., 2008). These reactions are carried out in the anhydrous ether solvents and under inert atmosphere. Of all the methods discussed, metathesis reactions appear to give highest yield and purity. In this study, development of a new approach for AB synthesis using struvite as a starting material is explored. This new synthetic route allows for recycling struvite, an otherwise wasteful product, to generate AB–a molecule with great potential for use as a stable hydrogen source.

## 2 Materials and methods

### 2.1 Reagents and equipment

Sodium borohydride (99%, Acros Organics), tetrahydrofuran (99.8%, Thermo Scientific), struvite (98%, Alfa Aesar), magnesium sulfate anhydrous (99.5%, Fisher Chemical), magnesium sulfate heptahydrate (98%, Thermo Scientific), sodium hydroxide (97%, Fisher Chemical), denatured ethanol (90% ethanol, 5% isopropanol, 5% methanol, Research Products International), and deuterium oxide (99.9% D, Cambridge Isotope Laboratories) were purchased from Fisher Scientific. Struvite from wastewater was prepared as described below. The NMR (^1^H and ^11^B) spectra were recorded using Bruker 400 MHz Avance III spectrometer. Perkin Elmer SpectrumOne IR spectrometer was used for FT-IR analyses. Thermogravimetric analysis and differential scanning calorimetry analysis were obtained using Mettler Toledo DSC3+ apparatus. Scanning electron microscope with energy dispersive spectrometry were recorded using ThermoScientific Prisma E scanning electron microscope.

### 2.2 Preparation of struvite from wastewater

Struvite crystals were prepared as previously reported (Witty et al., 2020). Briefly, one gallon (3.785 L) of human urine was gathered over several days and stored at 4°C. Then 10.501 g MgSO_4_·7H_2_O and 2.836 g NaOH was added and storage at 4°C was continued for 16 h. Crystals of struvite formed during this time were separated from the remainder of liquid waste by decanting and washing with water five times. The crystals were dried at 50°C to a freely flowing fine off-white powder. A small sample was resuspended in pure water and viewed using optical microscopy at ×40 magnification.

### 2.3 Preparation of ammonia borane from sodium borohydride and struvite

Struvite from two sources were used–the one prepared from wastewater and commercially available struvite purchased from Fisher Scientific. Different molar ratios of sodium borohydride (NaBH_4_) and struvite (NH_4_MgPO_4_·6H_2_O) were heated to the desired temperatures and stirred for 16–96 h. Briefly, sodium borohydride (NaBH_4_, 568 mg, 15 mmol) and struvite (NH_4_MgPO_4_·6H_2_O, 3.681 g, 15 mmol) were suspended in tetrahydrofuran (THF, 60 mL) as a slurry mixture in a 200 mL round bottom flask fitted with a reflux condenser and a magnetic stir bar. The reaction mixture was heated to 45°C and stirred for 72 h. Reaction completion was confirmed by ^11^B NMR when H_2_ production stops. After cooling to room temperature, the remaining solid (excess struvite and other by-products) was removed by filtration. The filtrate was then dried over anhydrous MgSO_4_ and filtered again. Finally, THF solvent was removed by rotary evaporation to obtain the white solid AB product (96 mg, 21%, >99% purity) without further purification. For the experiments that were halted before the completion of the reaction, crude product was purified using ethanol as follows. Crude AB was dissolved in ethanol and stirred for 10 min. After removing the solids by filtration, ethanol was removed by rotary evaporator to get purified AB product.

### 2.4 Scanning electron microscopy (SEM) and energy-dispersive X-ray spectroscopy (EDX)

Struvite crystals were analyzed by using ThermoScientific Prisma E scanning electron microscope in a charge-up reduction mode which required no sample coating. SEM image measurements were made with FEI xT software and EDX analyses were done by using Pathfinder X-ray microanalysis software.

### 2.5 Thermogravimetric (TG) and differential scanning calorimetry (DSC) analyses

Weight change (TGA) and differential heat flow (DSC) were simultaneous measured on dry samples using Mettler Toledo DSC3+ device. All measurement were performed under N_2_ atmosphere with a purge flow of 50 mL min^−1^. Approximately 10 mg of struvite was added to an open alumina crucible, heated from 30.0°C to 300.0°C at a rate of 20°C min^−1^ and then held at 300.0°C for another 1.5 min. AB samples were analyzed in open alumina crucibles under similar conditions using approximately 2 mg samples at a heating rate of 5°C min^−1^ until it reaches to 210°C.

### 2.6 FTIR and NMR analyses

Solid AB samples were analyzed directly on IR spectrometer equipped with diamond-ATR probe for identifying the product (Supplementary Figures S3–S8). For NMR, samples were dissolved in D_2_O solvent and analyzed by using Bruker 400 MHz Avance III spectrometer at room temperature. MNOVA software was used for spectral analysis. Ammonia borane signals: ^1^H NMR in D_2_O δ = 1.26 (q, 3H, BH_3_); ^11^B NMR in D_2_O δ = −24.11 (q, BH_3_).

## 3 Results and discussion

### 3.1 Physical and chemical compositions of struvite from wastewater

**FIGURE 1:**
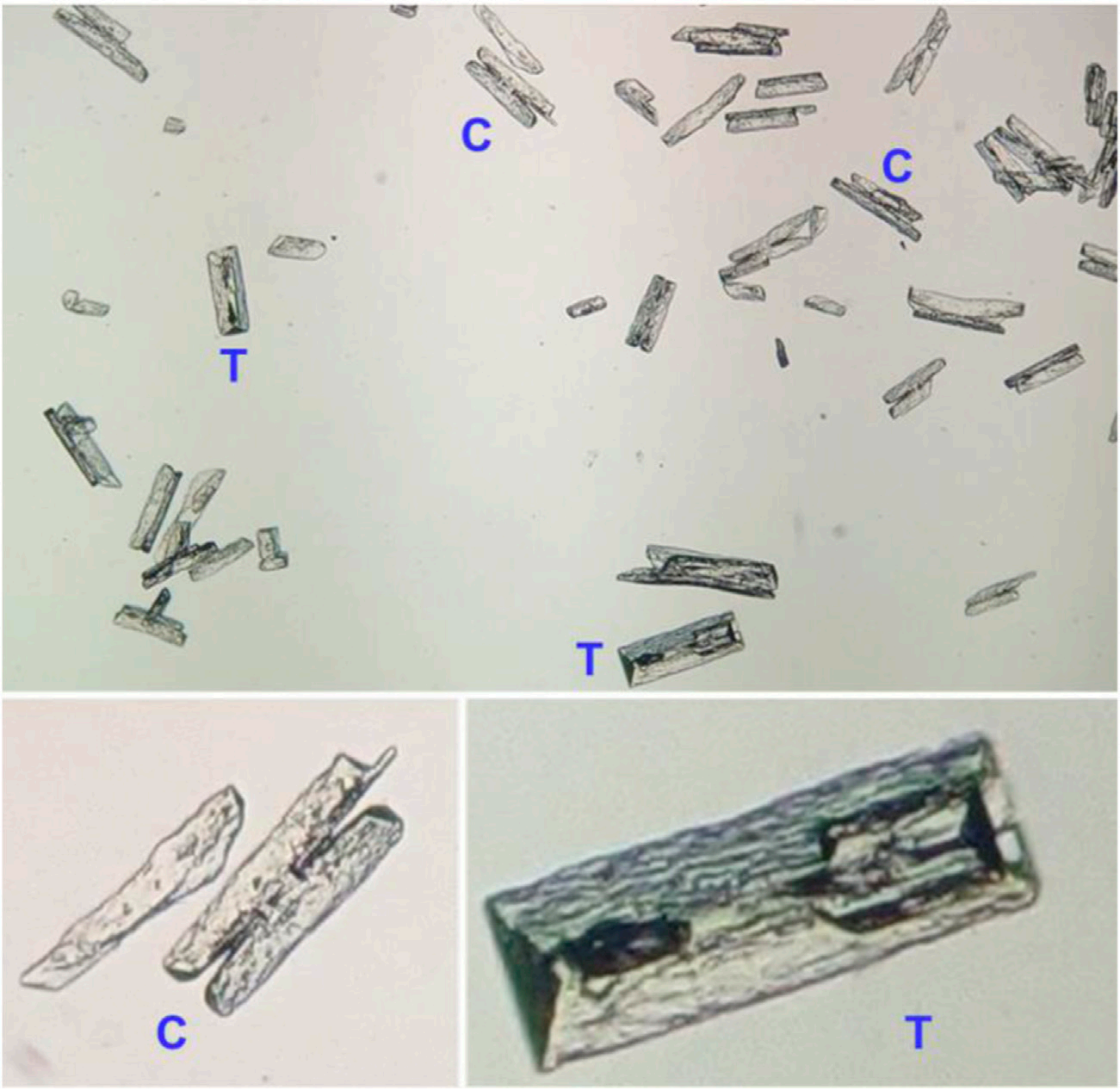
Optical microscopy of struvite crystals from human urine at 40x magnification. Triangular crystal faces (T) for Coffin Lid crystal forms are seen. Many other crystals have the Chromosome crystal form (C), some with crystal arms broken.

Struvite crystals from crude human urine were easily obtained. They were pitted as usual from formation in this complex mixture. However, they clearly showed the typical Coffin Lid form in some cases, as seen from the regular triangular crystal faces shown in Figure 1. Many other crystals were in the Chromosome form of struvite. This variability is caused by inconstant growth rates for the various crystal faces. Using inexpensive salts like MgSO_4_.7H_2_O or ash for base (Witty, 2016a) improve economic viability tremendously. Unlike many methods recommended for struvite production, no special energy input beyond labor is needed. Our methods also use optimal crystal growth for sedimentation and air drying.

**FIGURE 2:**
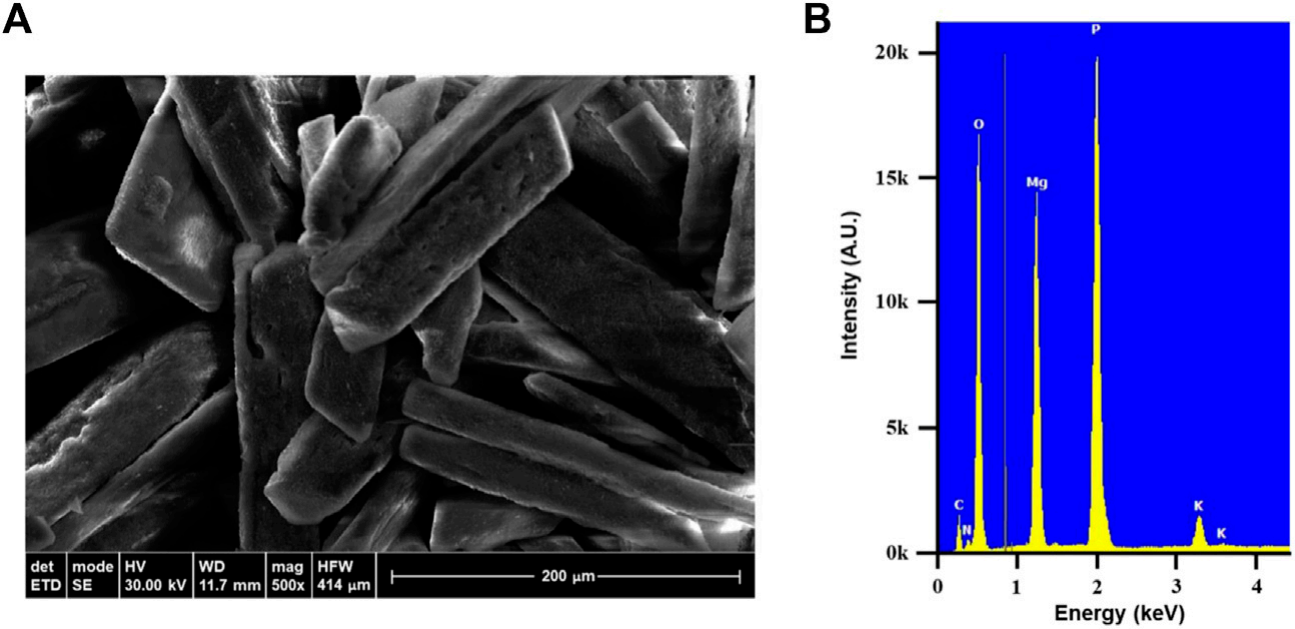
**(A)** SEM image of wastewater-derived struvite crystals. **(B)** EDX result of the struvite showing the presence of nitrogen, magnesium, phosphorous, oxygen, and potassium.

SEM image presented in Figure 2A shows both the Coffin Lid and Chromosome crystal forms with the average crystal length about 200 μm. Larger crystal size can be obtained by having fairly basic condition (pH 8–9) and allowing longer time for crystal growth. EDX result (Figure 2B) confirms the presence of struvite showing the abundant elements–N, Mg, P, and O. Struvite formula (NH_4_MgPO_4_·6H_2_O) has the N:P ratio of 1 to 1 but EDX result suggests this ratio to be 1 to 1.5. In addition to the typical elements from struvite, EDX also displays the presence of potassium (K). This likely comes from K-struvite (KMgPO_4_·6H_2_O) which is favored to form in higher pH values (Rodrigues et al., 2019) and may explain the discrepancy of the N:P ratio.

**FIGURE 3:**
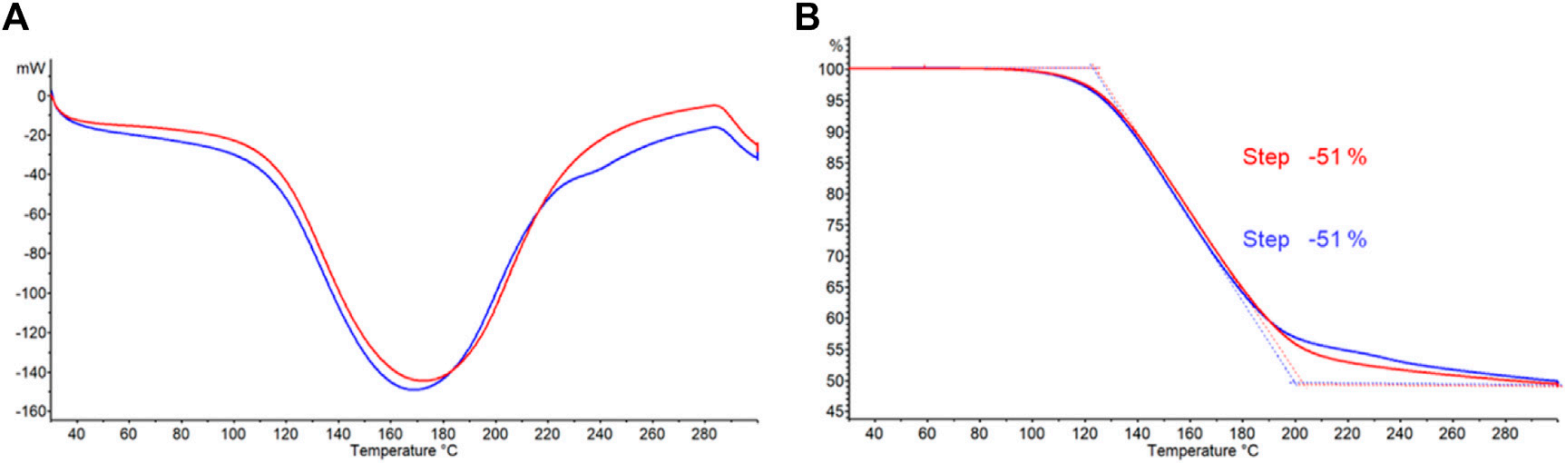
**(A)** Differential scanning calorimetry (DSC) and **(B)** thermogravimetric analysis (TGA) curves for struvite samples from different sources. Commercially available struvite is shown in blue and struvite from wastewater treatment is shown in red.

Differential scanning calorimetry (DSC) and thermogravimetric (TGA) analyses of commercially available struvite and struvite from wastewater treatment are shown in Figures 3A, B, respectively. Differential scanning calorimetry (DSC) data were simultaneously obtained along with respective thermograms shown. Previous studies suggested that decomposition of struvite depends on heating rate (Iqbal et al., 2008). In this study, struvite samples were heated at a rate of 20°C per minute. Both struvite samples starts decomposing at 60°C and the decomposition rates increased at 80°C with a major mass loss of approximately 43% between 110°C and 200°C. The total mass loss for both samples at 300°C was 51% (Figure 3B). This is consistent with the theoretical mass loss of 51.4% for losing 1 mol of ammonia and 6 mol of water leaving solid Mg-pyrophosphate (Bianchi et al., 2020). A single, broad endothermic peak in both samples (Figure 3A) corresponds to the major mass loss which occurs when water and ammonia are lost simultaneously from struvite. Almost identical thermograms and the amount of heat absorbed (Supplementary Figures S1, S2) for the mass losses from the two sources suggests that these two samples are essentially the same.

### 3.2 Synthesis of ammonia borane from struvite

Synthesis of ammonia borane has been widely reported using various different methods including liquid ammonia and ammonium salts as the sources of ammonia. Metathesis reaction of ammonium salts with metal borohydrides is usually performed under mild reaction conditions to give reasonable yields and purity (Ramachandran and Gagare, 2007; Heldebrant et al., 2008). Since the recycled struvite contains ammonia that can be liberated fairly easily, it is used as a source of ammonia in this metathesis reaction as shown in Eq. 1. The conditions and yields of AB synthesis experiments are summarized in Table 1. The influence on the yield by several factors were tested–the ratio of the two reactants, the variations in the amount of solvent and the temperature, and the reaction time.

$${\mathrm{NH}}_{4}{\mathrm{MgPO}}_{4}\cdot 6H_{2}O+{\mathrm{NaBH}}_{4}\;\to \;{\mathrm{NH}}_{3}{\mathrm{BH}}_{3}+{\mathrm{NaMgPO}}_{4}+H_{2}+6H_{2}O$$ (1)

**TABLE 1:** Varied conditions of ammonia borane synthesis from struvite and sodium borohydride. Commercially available struvite was used for most trials. Entry number 3, marked with *, is the struvite obtained from wastewater. ** crude yield with impurities.

Entry number	NaBH_4_ (mmol)	Struvite (mmol)	THF (mL)	Temperature (^o^C)	Time (hours)	Percent yield
1	15	15	30	45	72	19
2	15	15	60	45	72	21
3*	15	15	30	45	72	21
4	15	23	30	45	72	14
5	23	15	30	45	72	24
6	45	18	60	45	72	42
7	45	18	60	45	96	39
8	15	15	60	55	48	12
9	15	15	75	55	48	14
10	15	15	30	45	16	19**/**32**

Different ratios of sodium borohydride to struvite were examined first. Entry number 1 contains 1:1 ratio of the two reactants in 30 mL of THF solvent. Entry 2 uses the same ratio but doubling the volume of the solvent. Both were reacted at 45°C for 72 h and yielded similar amounts of product at 19%–21%. Using struvite obtained from wastewater (entry 3) produced results no different to that from commercial struvite (entry 1). Results from entry 1 and 3 confirm the DSC and TGA analyses from Figure 3 that the quality of the two starting materials is essentially the same. Observing gas evolution is a good way to monitor completion of the reaction as the hydrogen gas cease to form when NaBH_4_ is completely consumed. In order to see if the reaction time can be reduced, reaction temperature was increased to 55°C as seen in entry 8 and 9. At this temperature, the reaction was completed in 48 h. However, raising the temperature reduced the yield to 12%–14% which indicates that AB product decomposition was also taking place as it was being made. AB is known to be stable in the solid state but undergoes slow decomposition in solvents such as THF and the decomposition of AB is expedited at temperatures beyond 50°C (Wang and Geanangel, 1988). In addition, NaBH_4_ breaksdown at a faster rate at higher temperatures (Schlesinger et al., 1953) making it less available to react with struvite.

The NaBH_4_ to struvite ratio was then changed to 1:1.5 and 1.5:1 in entry 4 and 5 respectively. When struvite was in excess, we observed a drop in the yield to only 14%. On the other hand, the yield increases to 24% when extra NaBH_4_ was added in the reaction. This could be rationalized by the fact that struvite crystal contains 6 mol of water per 1 mol of ammonium, and water also reacts with borohydride producing hydrogen gas. In fact, these 6 mol of water will consume 1.5 mol of NaBH_4_. This suggests that instead of 1 mol of borohydride for 1 mol of struvite, 2.5 mol of borohydride is consumed. To confirm this assumption, a 2.5 to 1 mol ratio of borohydride and struvite was tested in trials 6 and 7. Increasing NaBH_4_ yielded higher amounts of products at a 39%–42% range. Because NaBH_4_ readily reacts with water from struvite, having excess of it will ensure that the reaction moves forward to form ammonia borane. Metathesis reactions using borohydride and ammonium salts are generally carried out under mild temperatures and at low reagent concentrations. In fact, the product yield and purity depend on the reaction condition kept at low concentrations (Ramachandran and Gagare, 2007). We also tried different concentrations in our experiments increasing the solvent amount used for the reactions but there are negligible differences in the yield or the purity.

Examination of the mole ratio variability suggests that higher yields can be achieved with excess NaBH_4_. The best result which gives roughly 42% yield is obtained from NaBH_4_ to struvite ratio of 3:1 at low temperature. In practical applications, the wastewater product, struvite, should be the one present in excess amount. Nevertheless, even with the stoichiometric ratio of the reactants, 20% yield is obtained at a high purity. The THF solvent from this reaction can be reclaimed from the rotary evaporator minimizing the waste. Further improvement on the yield in the future can be made by incorporationg anhydrous reagents in the reaction mixture to absorb water from struvite crystals.

### 3.3 Analysis of ammonia borane product for purity

**FIGURE 4:**
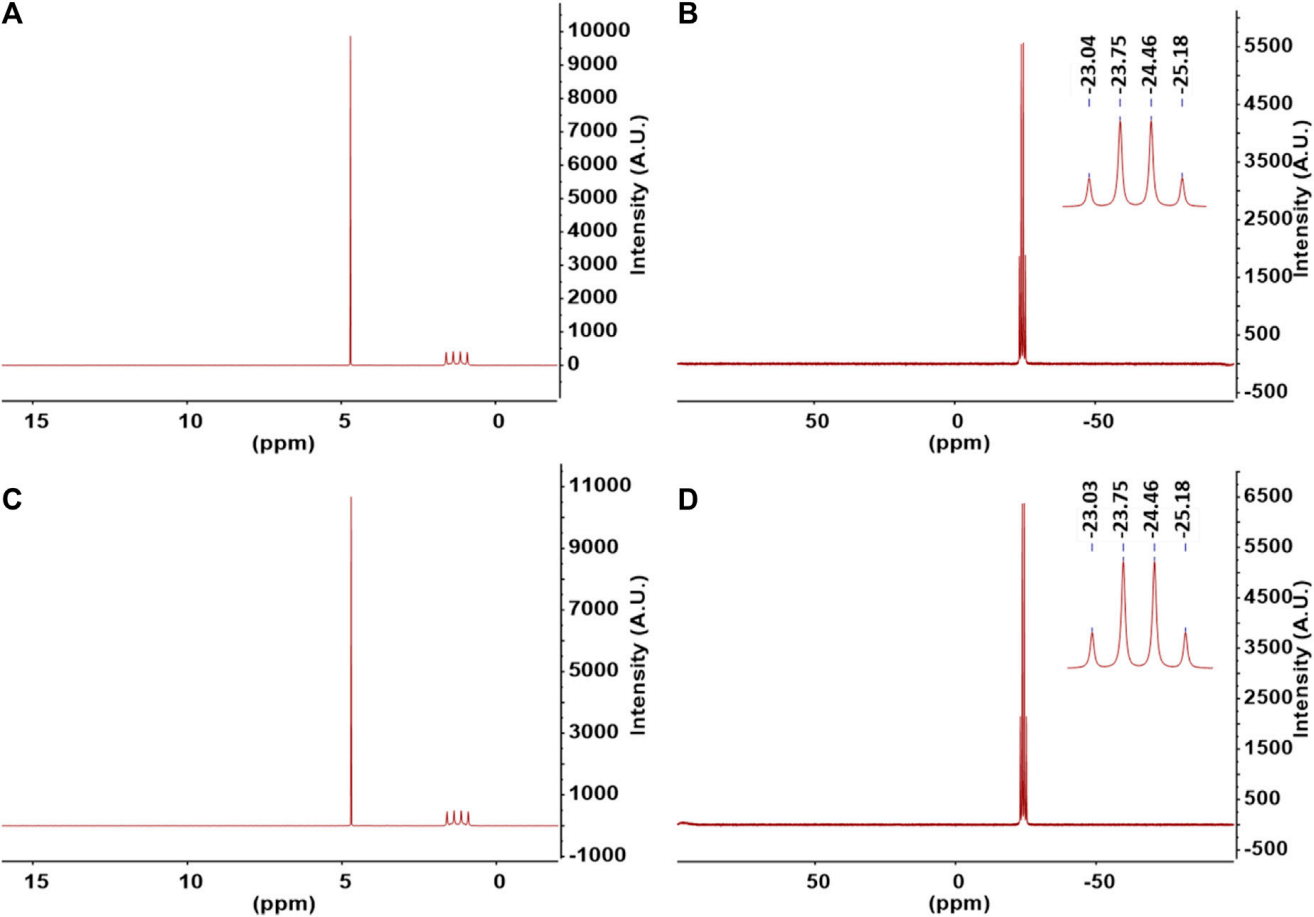
**(A)** ^1^H NMR and **(B)** ^11^B NMR of ammonia borane synthesized using commercially available struvite. **(C)** ^1^H NMR and **(D)** ^11^B NMR of ammonia borane synthesized using reclaimed struvite from wastewater. Samples were dissolved in D_2_O and spectra acquired at room temperature.

In order to analyze the purity of ammonia borane product, nuclear magnetic resonance (NMR) spectroscopy was employed. The samples were dissolved in D_2_O solvent for the analysis. This solvent was chosen since the primary impurities such as boric acid and borates dissolve in water but do not dissolve in standard NMR solvents including DMSO-d_6_, THF-d_4_, or CH_3_CN-d_3_. Therefore, we expect any impurities present in the sample to appear on the spectra when dissolved in D_2_O. ^1^H and ^11^B NMR of ammonia borane synthesized from commercially available struvite and struvite from wastewater are shown in Figures 4A–D, respectively. ^1^H NMR spectra of AB products in Figures 4A, C exhibit a quartet of BH_3_ at δ1.26 ppm (J = 92.0 Hz) as a result of ^11^B nucleus splitting the proton signal. A quartet peak at δ-24.11 ppm in the ^11^B NMR spectrum (Figures 4B, D) indicates the presence of a boron atom with three hydrogens as in -BH_3_. Both ^11^B and ^1^H NMR spectra in D_2_O show only the peaks attributed to AB product and HDO solvent residue (^1^H NMR δ4.70 ppm) from proton exchange with NH_3_. The major concern when using borohydrides with even a trace amount of water in the reaction is the reactivity of boron towards water producing undesired side products such as boric acid and tetrahydroxyborate [B(OH)_4_⁻] species (Schlesinger et al., 1953). There are no traces of the reactant NaBH_4_ or other boron species on ^11^B NMR as well as other hydrogen-containing compounds in the ^1^H NMR spectra. Once the reaction is allowed to continue until completion, ammonia borane produced from this process, without further purification, is of very high purity at >99% as determined by ^1^H and ^11^B NMR spectroscopy.

In cases where the reaction is stopped before completion, the product contains high amounts of impurities. Example sample (Table 1 entry 10) was prepared to test if simple purification method can eliminate impurities and give a pure AB product. The majority of impurities present appears to be borate (12%) and unreacted borohydride (4%) (Supplementary Figure S9E). After dissolving crude product in ethanol, filtering the impurities, and evaporating the solvent, purified product was achieved. This simple purification step provides pure AB product as confirmed by spectroscopic analyses (Supplementary Figures S6, S9C, D). The yield after purification is 19% which is essentially the same as the yield obtained when the reaction is allowed to continue until completion at 72 h. It is reasonable to assume that the ongoing decomposition of the AB product as it is being made limits the amount of product we can recover. Synthetic routes that provide impure AB product present major challenges for purification as AB must be of high purity for it to be useful as a hydrogen fuel. One of the methods used to improve the purity of AB involves dissolving the crude AB in the organic solvents such as ether and extracting the impurities with highly basic aqueous sodium hydroxide solution (Drost, 2014). Our method uses ethanol which is a common, inexpensive solvent and does not require extraction using caustic solutions. Borohydride in crude product is easily removed by reaction with ethanol. The resulting product, a form of borate, is insoluble in ethanol. By filtering out the insoluble impurities, we achieve a pure AB at >99% purity.

### 3.4 Thermolytic properties of ammonia borane by thermogravimetric analysis

**FIGURE 5:**
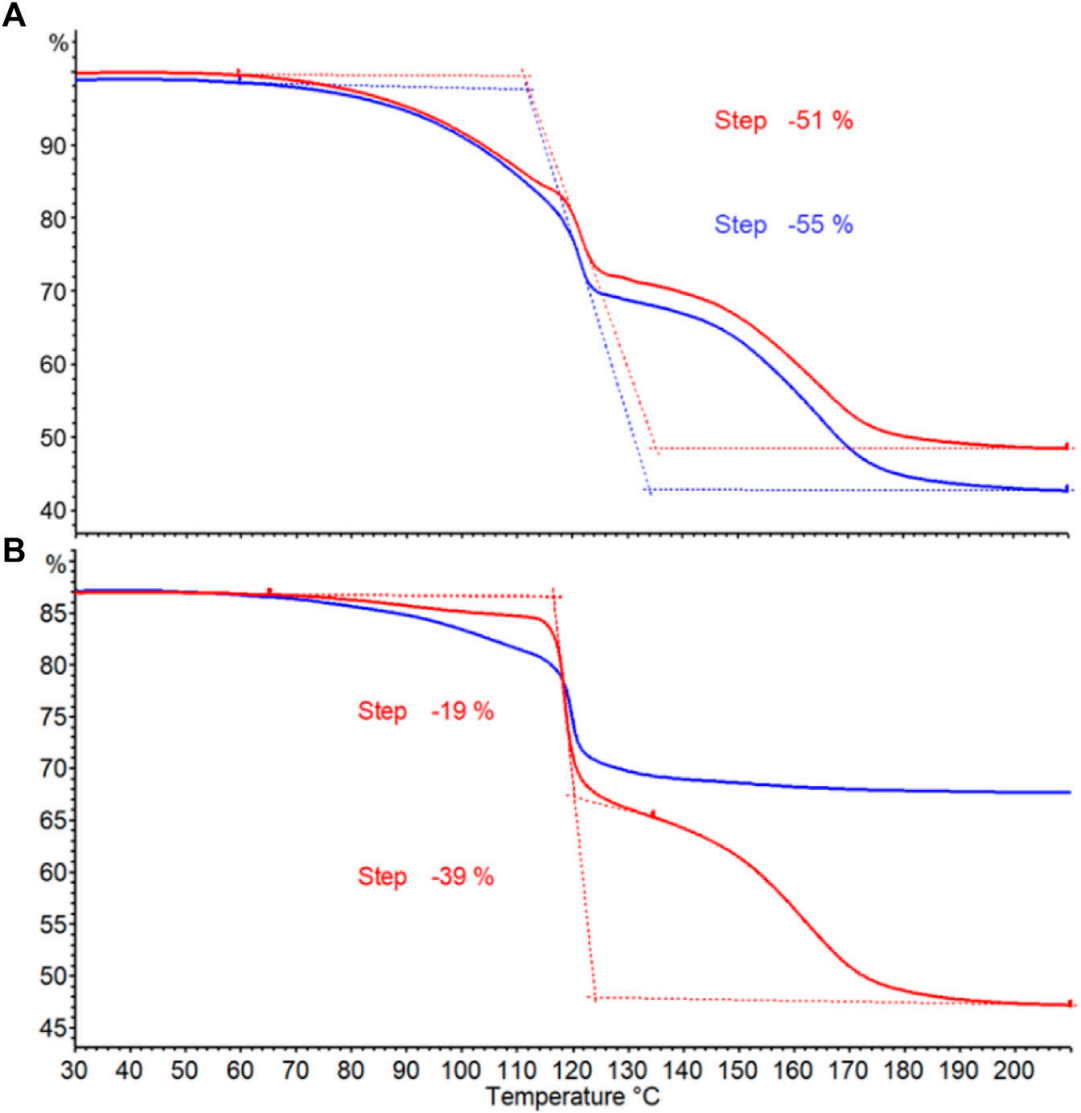
Thermogravimetric curves for determining thermal decomposition of AB. **(A)** Pure AB products from wastewater struvite (blue) and commercial struvite (red). **(B)** AB sample obtained from stopping the reaction at 16 h (Table 1 entry 10). Crude AB (blue) and purified AB (red) obtained from ethanol purification.

Thermogravimetric (TG) analysis is routinely used for screening hydrogen storage materials including ammonia borane. Thermal decomposition of ammonia borane occurs in two stages. The first stage involves AB decomposition into hydrogen gas and aminoborane (NH_2_BH_2_). At a higher temperature, AB decomposes in the second stage to hydrogen gas as well as ammonia (NH_3_), diborane (B_2_H_6_), and borazine (B_3_N_3_H_6_) (Frueh et al., 2011; Demirci, 2021). TG curves obtained from AB products prepared are shown in Figure 5. The onset temperature of the first main decomposition is found consistently around 65°C for all the samples. These onset temperatures are comparable to those of AB doped with metal chlorides and of AB in mesoporous materials (Nakagawa et al., 2016; Sullivan et al., 2017). The onset temperature for the second stage decomposition is 135°C for all pure samples. Crude AB with impurities does not show second stage decomposition (Figure 5B blue line). Percent weight losses for pure AB products from wastewater struvite and commercial struvite are 55% and 51% respectively (Figure 5A). Purified AB also shows 39% weight loss while impure AB only loses 19% (Figure 5B). The hydrogen content in AB is only 19.6 wt%, yet the extra weight loss come from losing decomposition by-products such as NH_2_BH_2_, NH_3_, B_2_H_6_, and B_3_N_3_H_6_. This higher weight loss is due to the use of open crucible which allows the gases to escape easily therefore preventing the reaction of by-products towards the formation of polymeric solid residues (Petit and Demirci, 2019). Likewise, another synthesis stopped at 24 h and subjected to ethanol purification also show similar TGA analyses for crude and purified AB (Supplementary Figure S14). We observe that while the weight losses and the curves for pure AB products from one-step synthesis that requires no purification are similar (Figure 5A), they are slightly different from those of ethanol-purified AB. The curve at the first stage of decomposition is sharper (Figure 5B) and the weight loss is less for purified AB. Varying degree of crystallinity in AB products obtained from different solvents may be responsible for the differences in the TG profiles observed.

## 4 Conclusion

Hydrated crystal struvite (NH_4_MgPO_4_·6H_2_O) from wastewater is used in this research for the synthesis of ammonia borane (NH_3_BH_3_). The chemical nature of ammonia borane as a source of hydrogen makes it valuable for potential use in applications where a compact and safe hydrogen storage is required. Future direction includes scaling up the process and mitigating the challenges that come from safely handling the hydrogen gas byproduct. Human urine contains many chemical entities, proteins and even human and bacterial cells. It is remarkable to have such a simple process that starts with a high degree of chemical and biological complexity and ends up with such a high degree of purity for ammonia borane that is used as a renewable energy source. This process is extremely valuable for wastewater and energy industries as something that is considered a waste product is converted into a valuable material.

## Data Availability

The original contributions presented in the study are included in the article/Supplementary Material, further inquiries can be directed to the corresponding author.
