# Supplementary material for: Transformation of struvite from wastewater to a hydrogen fuel storage compound ammonia borane
Source: Front Chem. 2023 Nov 7;11:1269845. doi: 10.3389/fchem.2023.1269845 (PMC10662098; doi:10.3389/fchem.2023.1269845)
Supplement: Supplementary file 1 [file DataSheet1.pdf]

Supplemental Information

Transformation of struvite from wastewater to a hydrogen fuel storage compound ammonia borane

Nin Dingra[1], Michael Witty[3], Marie Celis[1], Narendra Boppana[2], Theppawut Ayudhya[1*]

[1] Department of Chemistry, University of Texas Permian Basin, Odessa, Texas, USA.
[2] Department of Chemical Engineering, University of Texas Permian Basin, Odessa, Texas, USA.
[3] School of Pure and Applied Sciences, Florida SouthWestern State College, Fort Myers, Florida, USA.

*** Correspondence:**
Theppawut Ayudhya
ayudhya_t@utpb.edu

Table of Contents

Contents	Page
Table of Contents	S1
Thermal analysis of struvite samples by differential scanning calorimeter	S2
IR spectrum of ammonia borane (Table 1 entry #1)	S3
IR spectrum of ammonia borane (Table 1 entry #3)	S4
IR spectrum of ammonia borane (Table 1 entry #10) before purification	S5
IR spectrum of ammonia borane (Table 1 entry #10) after purification	S6
IR spectrum of ammonia borane (reaction stopped at 24 hours) before purification	S7
IR spectrum of ammonia borane (reaction stopped at 24 hours) after purification	S8
^{1}H and ^{11}B NMR spectra of ammonia borane (Table 1 entry #10) before and after purification	S9
^{1}H NMR spectrum of crude product (reaction stopped at 24 hours) before purification	S10
^{11}B NMR spectrum of crude product (reaction stopped at 24 hours) before purification	S11
^{1}H NMR spectrum of ammonia borane (reaction stopped at 24 hours) after purification	S12
^{11}B NMR spectrum of ammonia borane (reaction stopped at 24 hours) after purification	S13
TGA of crude and purified ammonia borane (reaction stopped at 24 hours)	S14

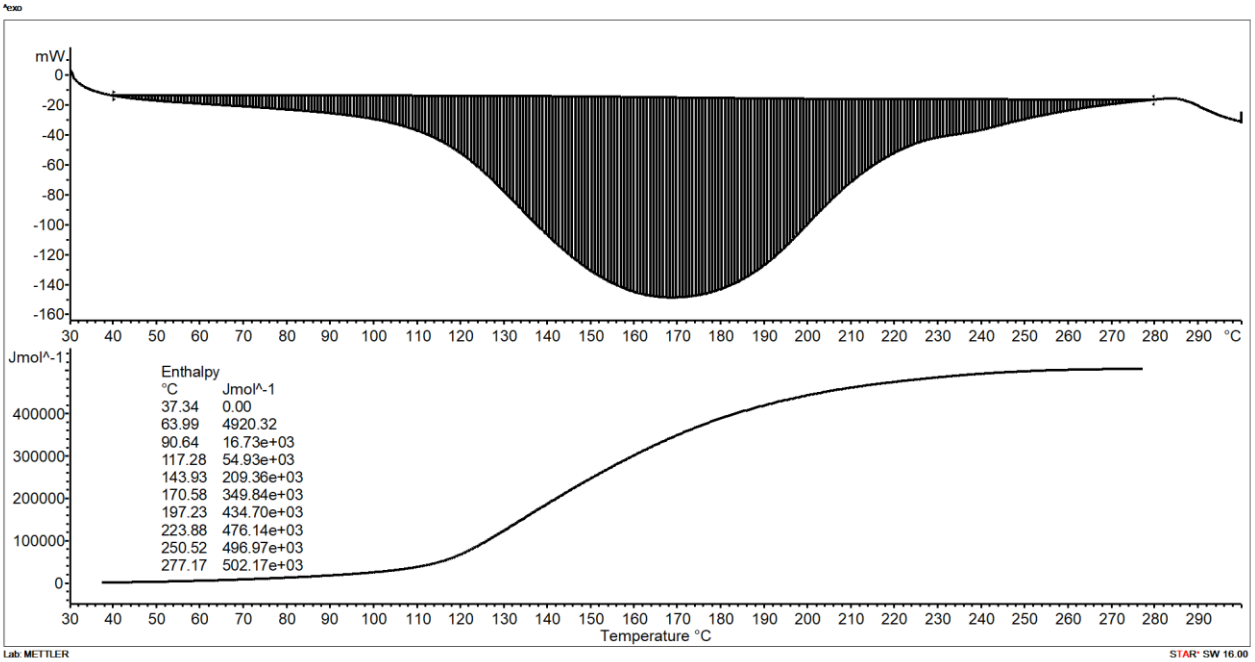

Figure S1. DSC curve of struvite purchased from Sigma-Aldrich. Only a single, broad endothermic peak is visible. This corresponds to the major mass loss during heating. The presence of a single peak in the DSC curve indicates that water and ammonia are lost simultaneously from the struvite structure.

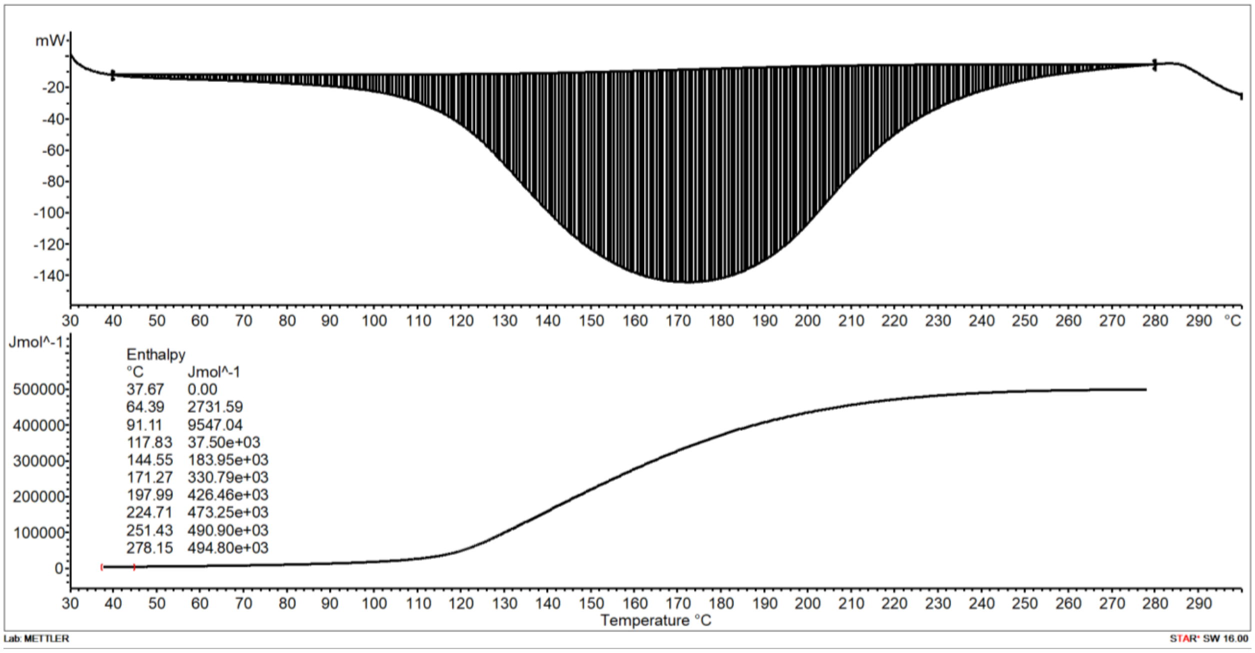

Figure S2. DSC curve of struvite prepared from wastewater. It is almost identical to the commercially available one from Fig S1.

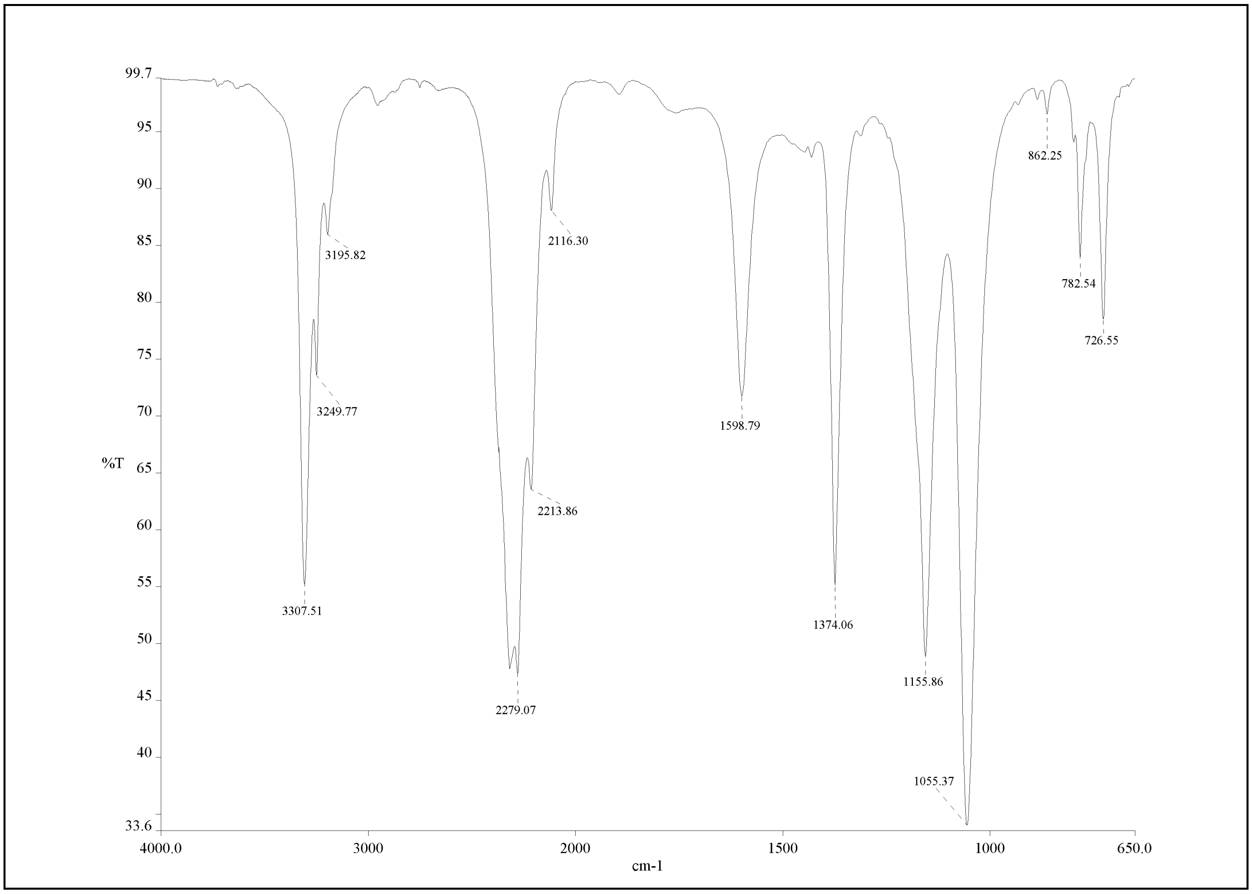

Figure S3. IR spectrum of ammonia borane synthesized using struvite from Sigma-Aldrich (Table 1 trial #1). The bands are attributed to the different vibrational modes of the molecule. Asymmetric and symmetric N-H stretch (3307 and 3249 cm^{-1}), asymmetric B-H stretch (2279 cm^{-1}), asymmetric and symmetric N-H def (1598 and 1374 cm^{-1}), asymmetric and symmetric B-H def (1155 and 1055 cm^{-1}), B-N stretch (782 cm^{-1}).

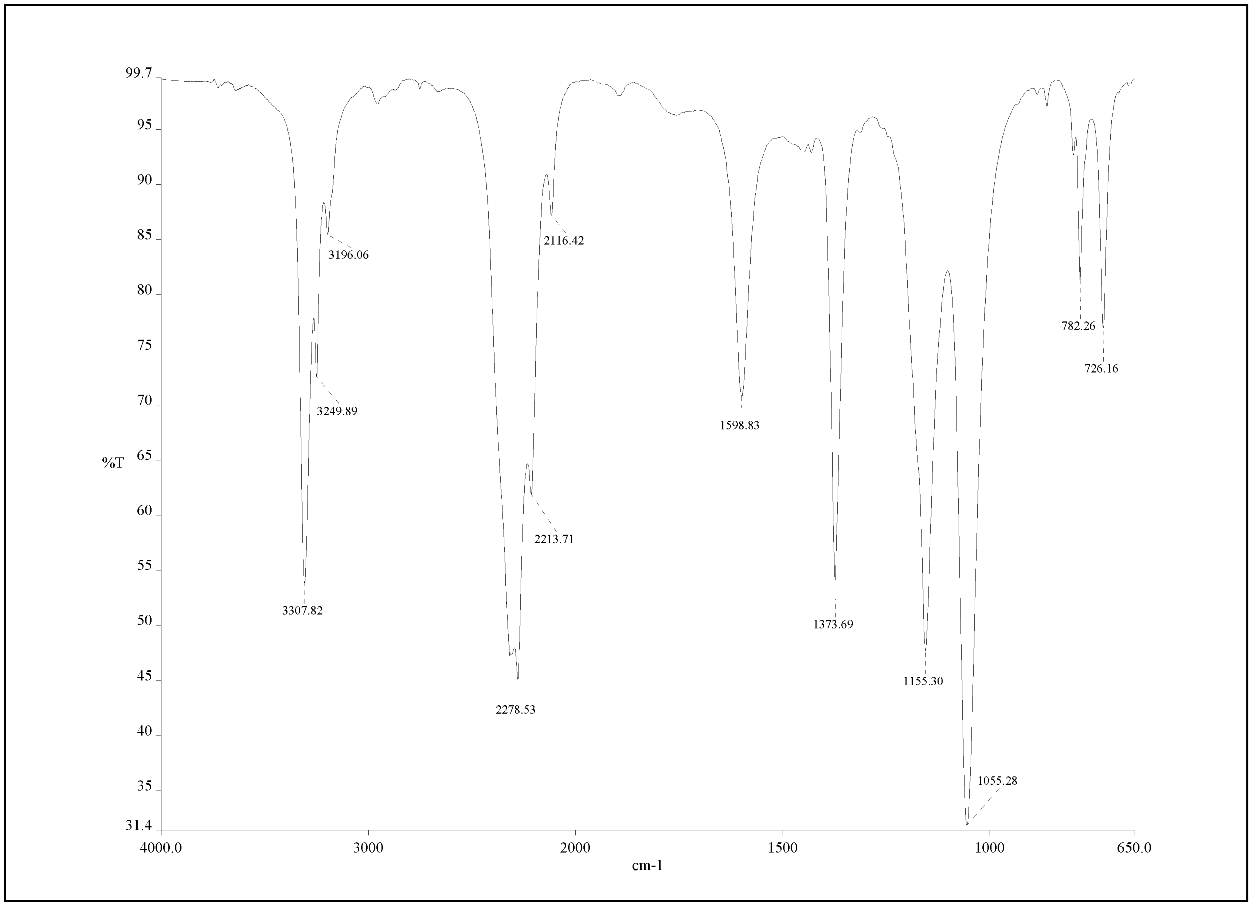

Figure S4. IR spectrum of ammonia borane synthesized from wastewater-derived struvite (Table 1 trial #3). The bands are almost identical to those of Fig S3.

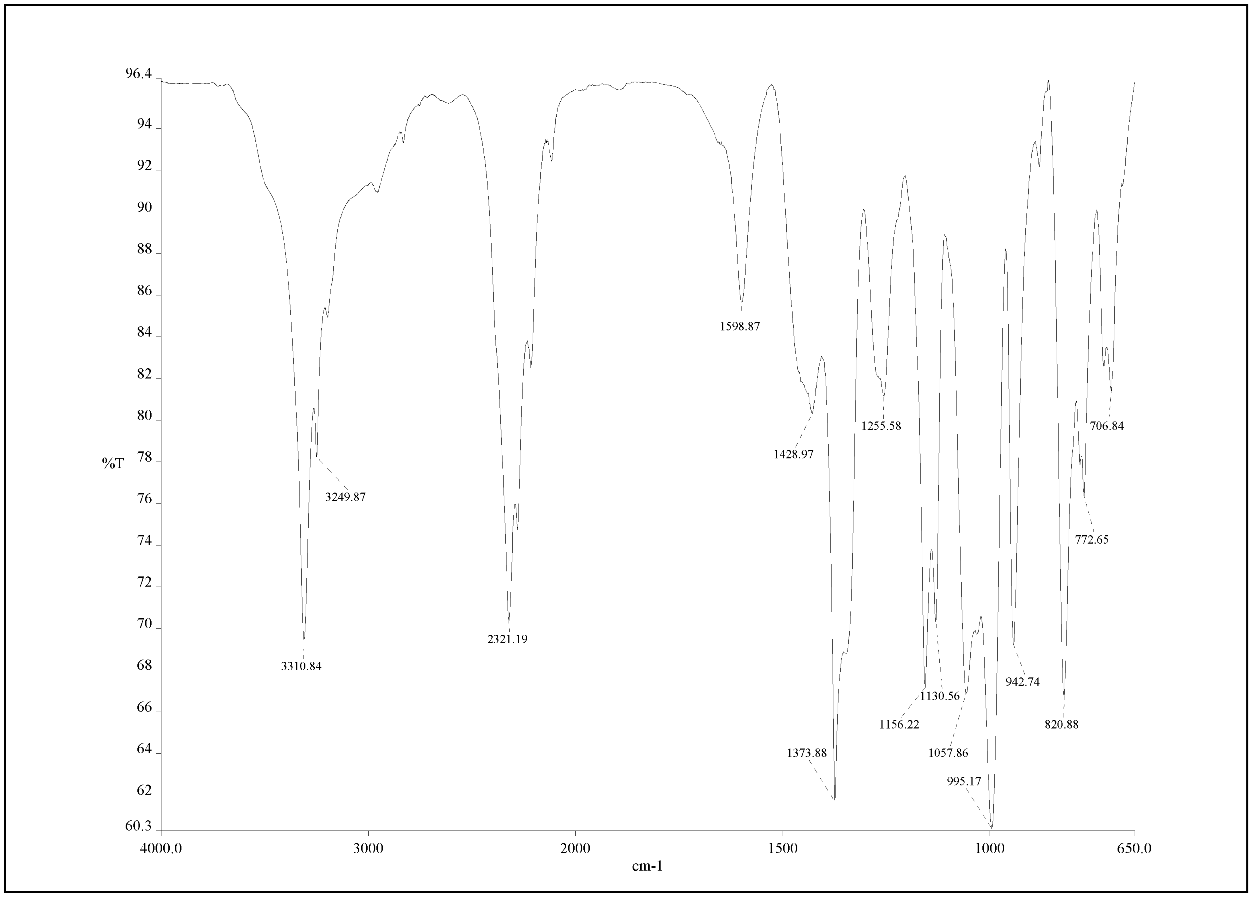

Figure S5. IR spectrum of crude ammonia borane synthesized using commercially available struvite from the reaction stopped at 16 hours (before purification).

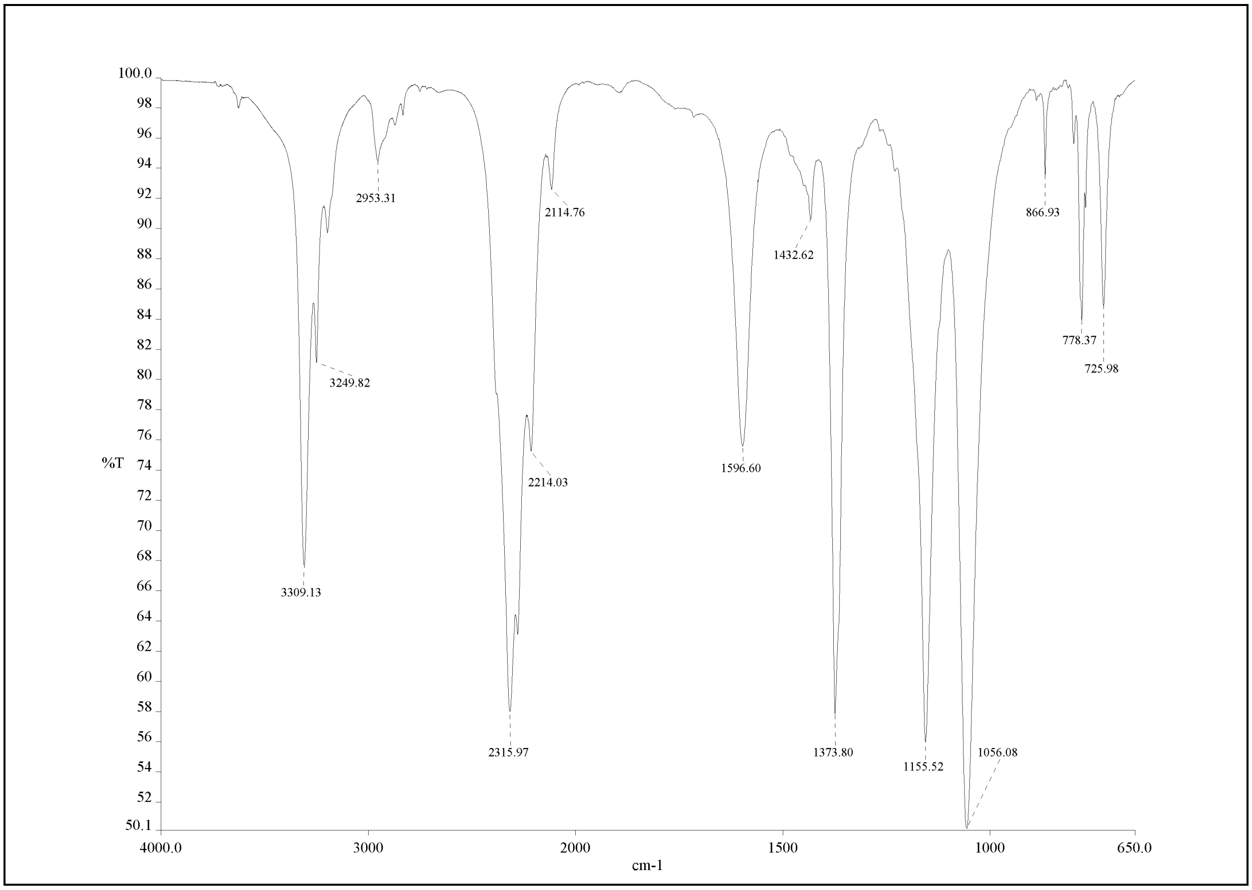

Figure S6. IR spectrum of ammonia borane synthesized using commercially available struvite from the reaction stopped at 16 hours (after purification). The bands are almost identical to those of Fig S3 and Fig S4.

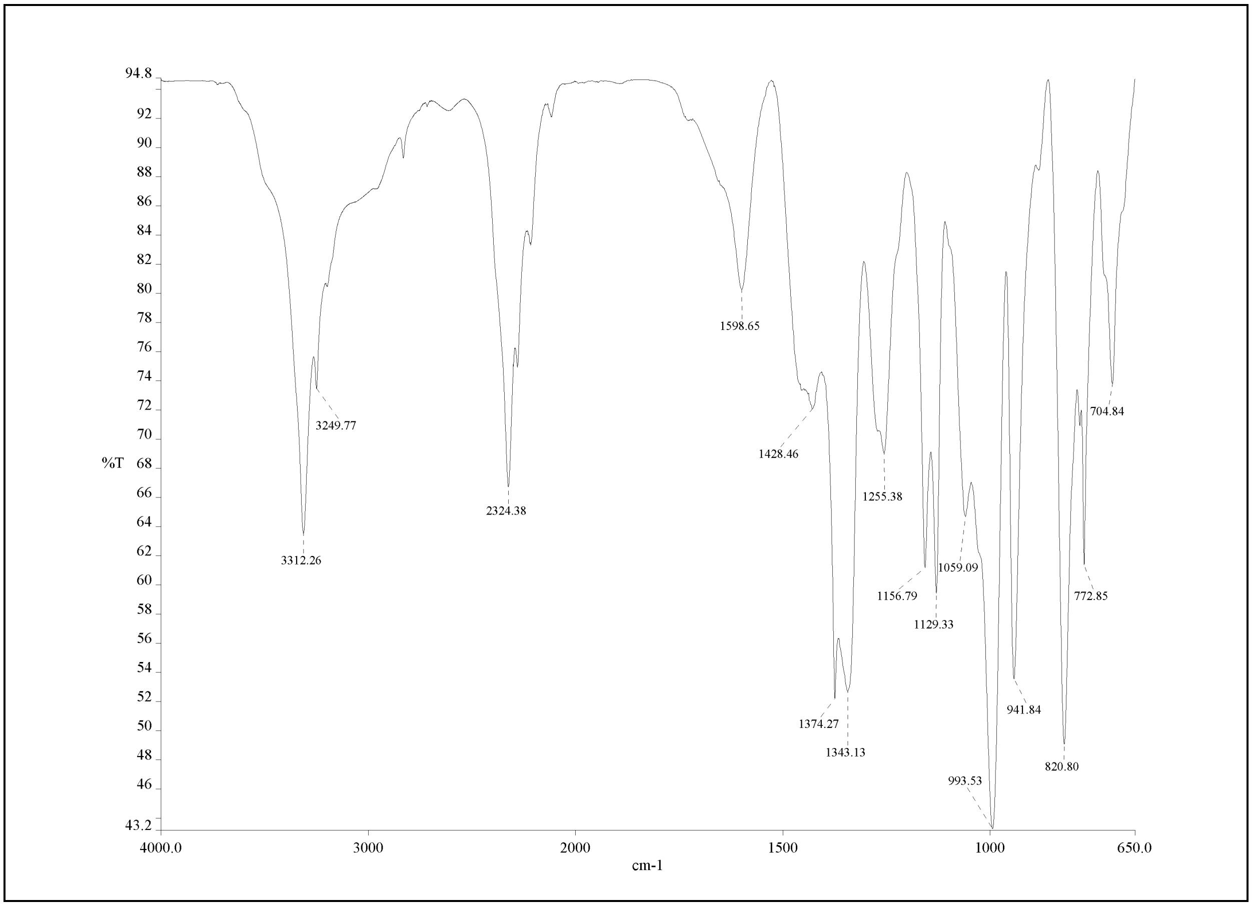

Figure S7. IR spectrum of ammonia borane synthesized using commercially available struvite from the reaction stopped at 24 hours (before purification).

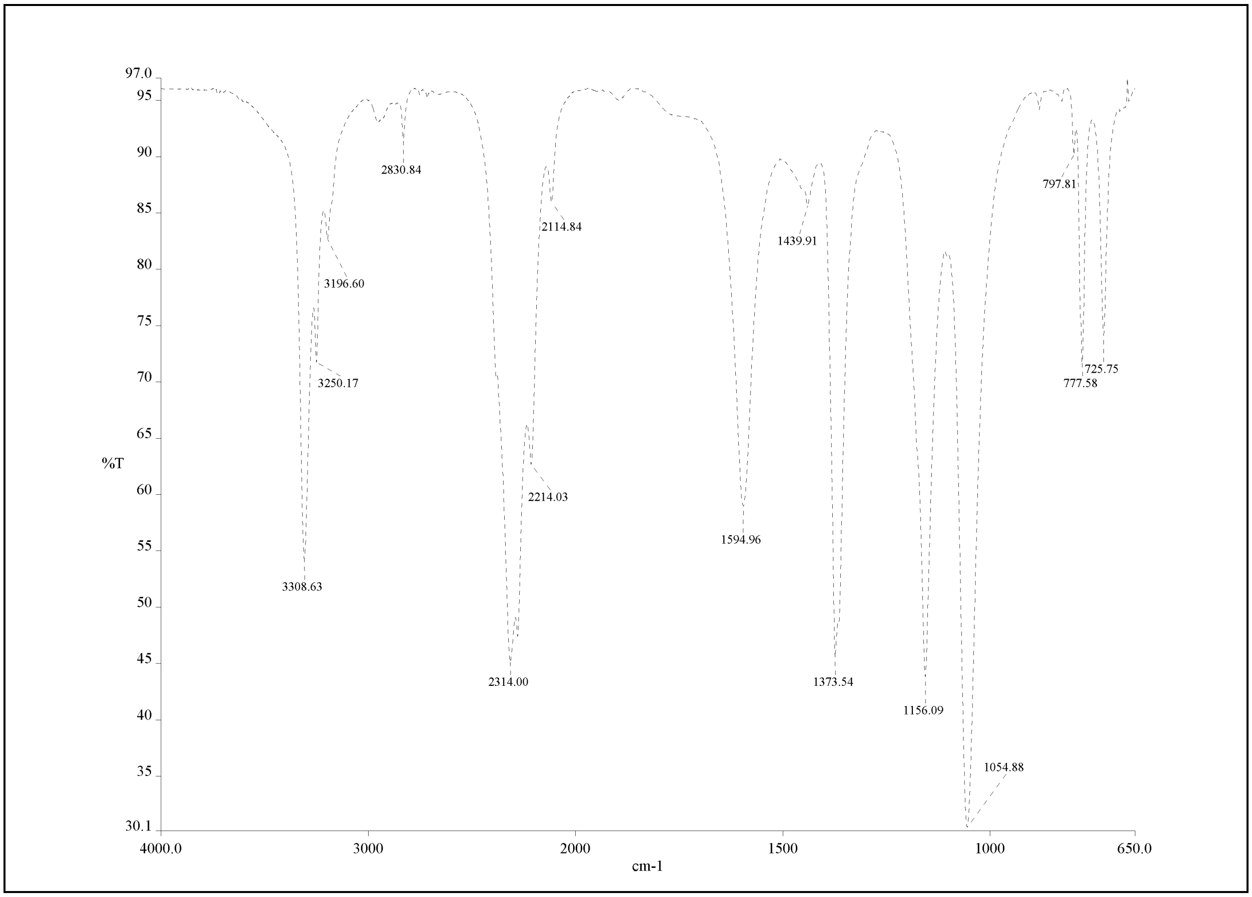

Figure S8. IR spectrum of ammonia borane synthesized using commercially available struvite from the reaction stopped at 24 hours (after purification).

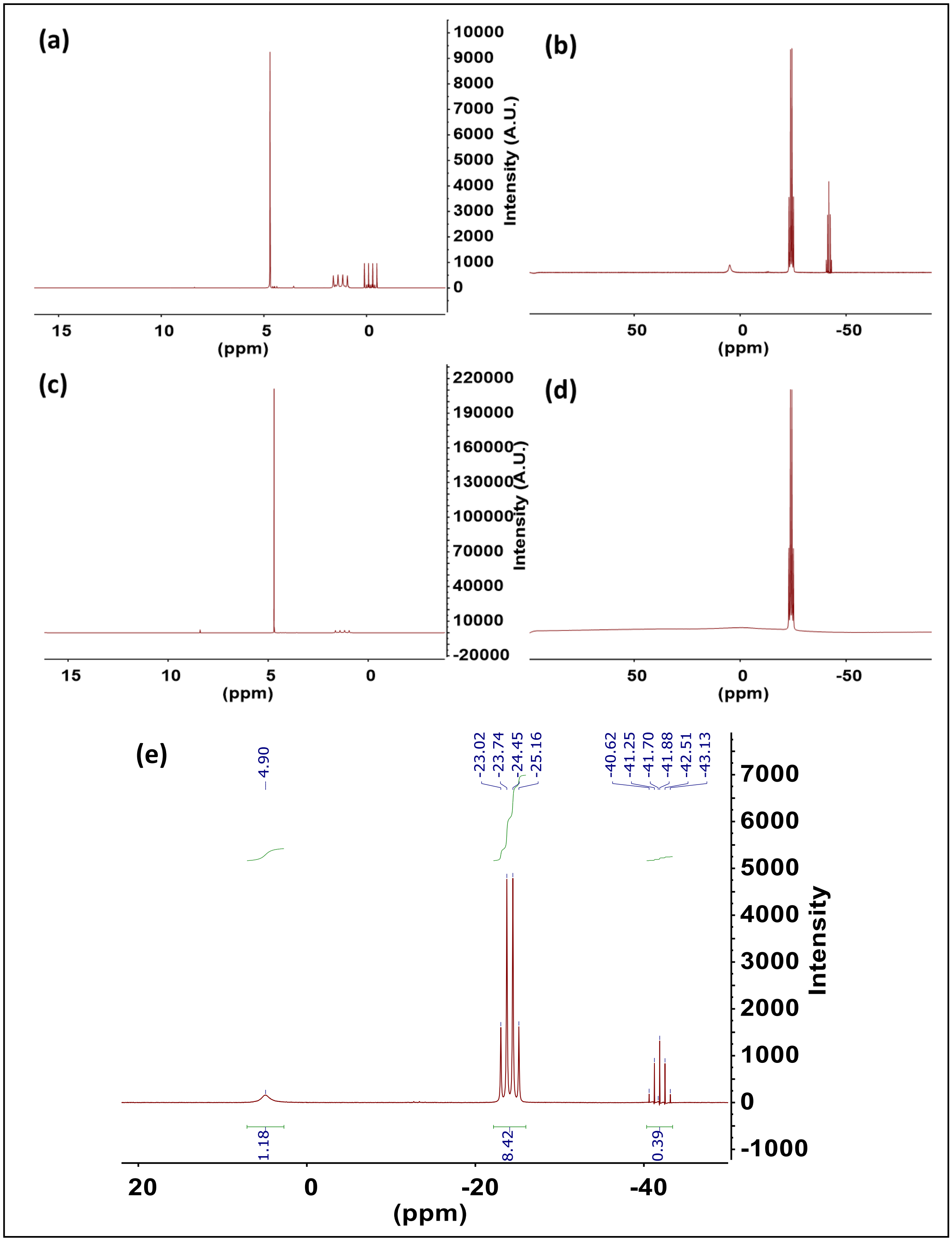

Figure S9. (a) ^{1}H NMR and (b) ^{11}B NMR of crude AB from the reaction stopped at 16 hours (c) ^{1}H NMR and (d) ^{11}B NMR of purified AB product after ethanol purification (e) ^{11}B NMR of impure AB from the reaction stopped at 16 hours with integrations to identify and calculate the impurities. A peak at δ4.90 represents borates and the peaks at δ-41 represent borohydride. Samples were dissolved in D_2O and spectra acquired at room temperature.

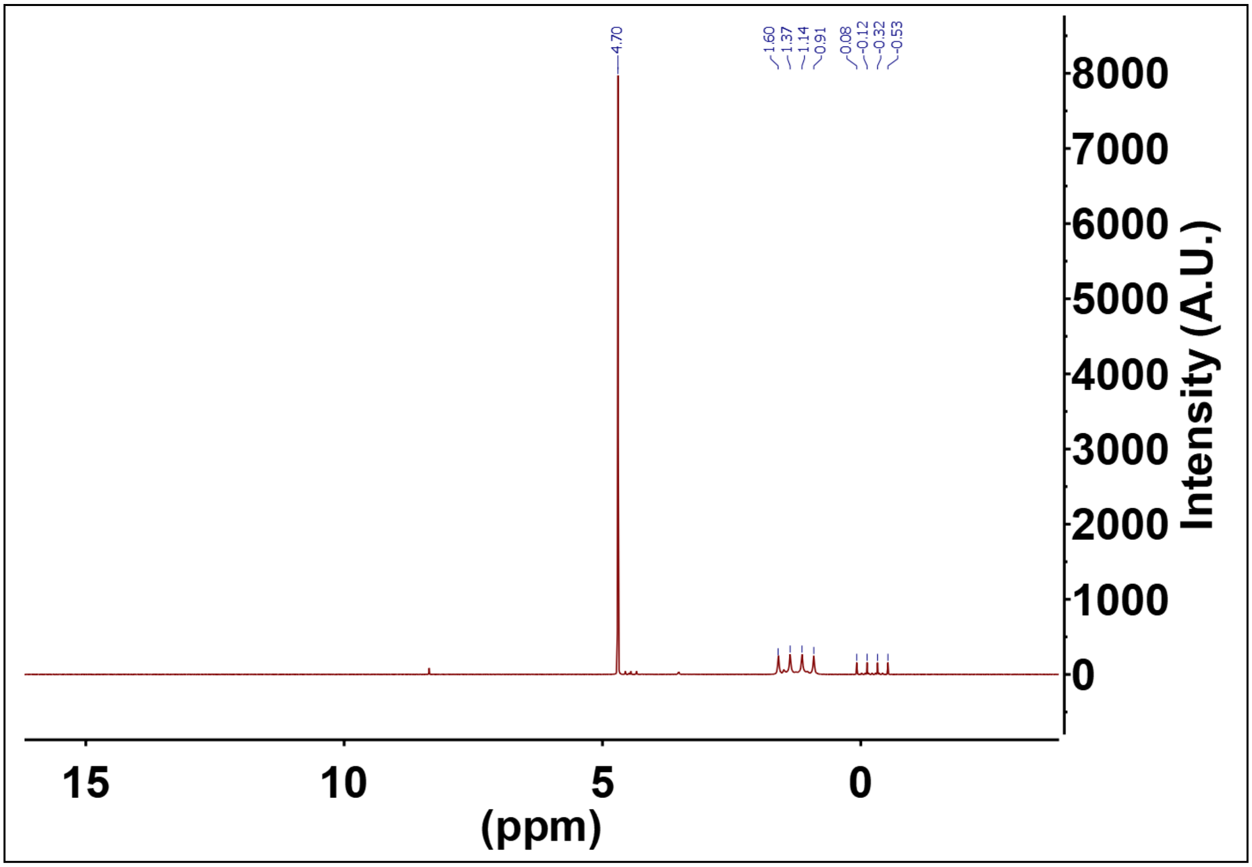

Figure S10. ^{1}H NMR spectrum of crude ammonia borane (before purification) synthesized using commercially available struvite from the reaction stopped at 24 hours. Crude yield is 37%.

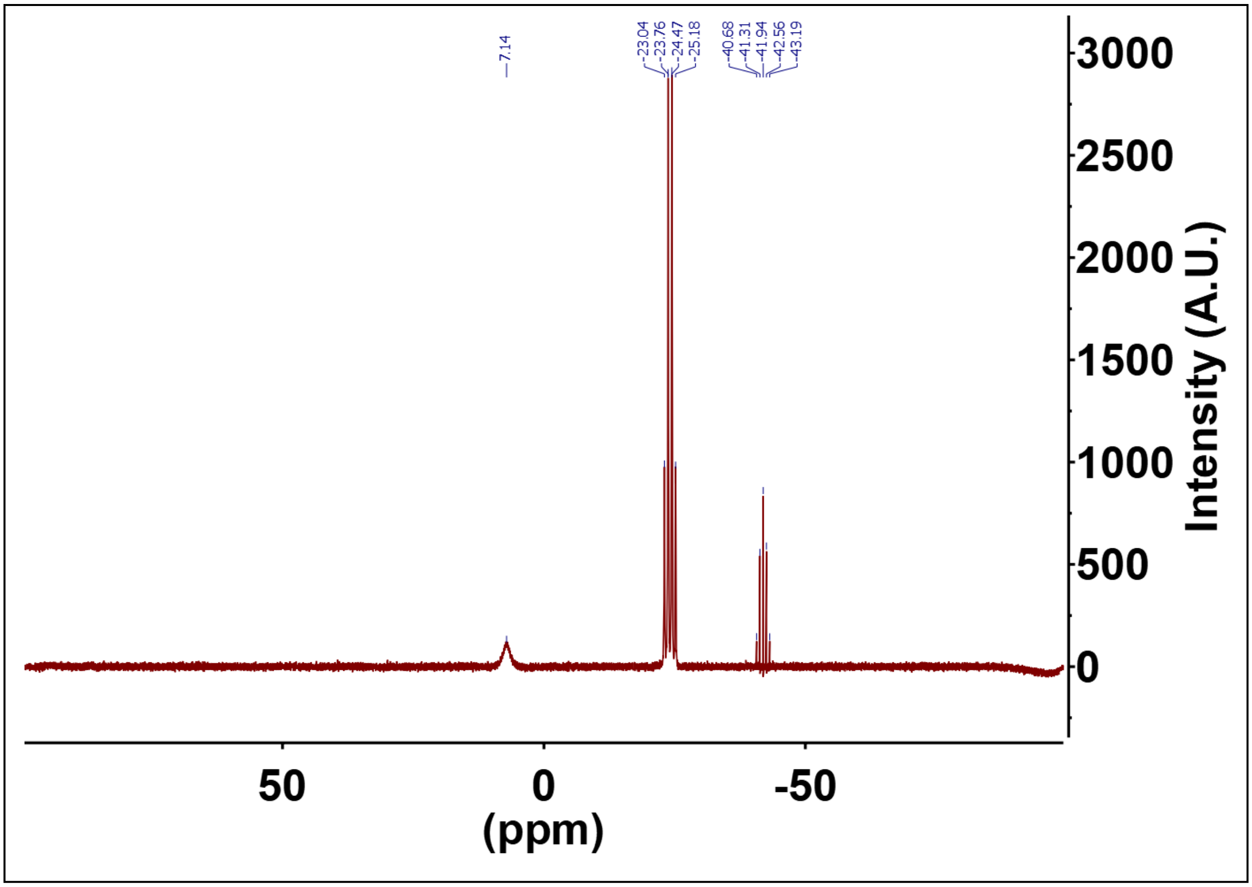

Figure S11. ^{11}B NMR spectrum of crude ammonia borane (before purification) synthesized using commercially available struvite from the reaction stopped at 24 hours. Crude yield is 37%.

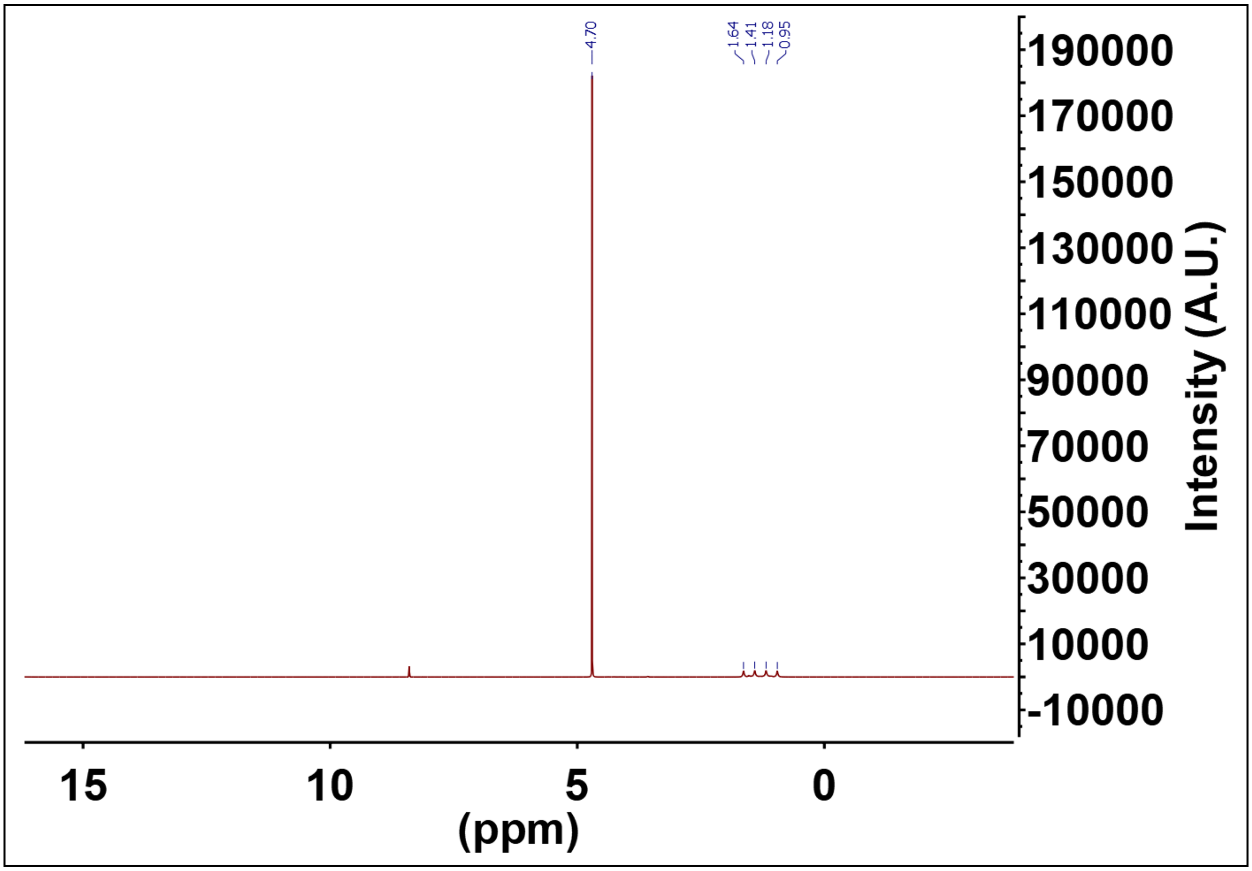

Figure S12. ^{1}H NMR spectrum of ammonia borane (after purification) synthesized using commercially available struvite from the reaction stopped at 24 hours (after purification). Percent yield is 21%.

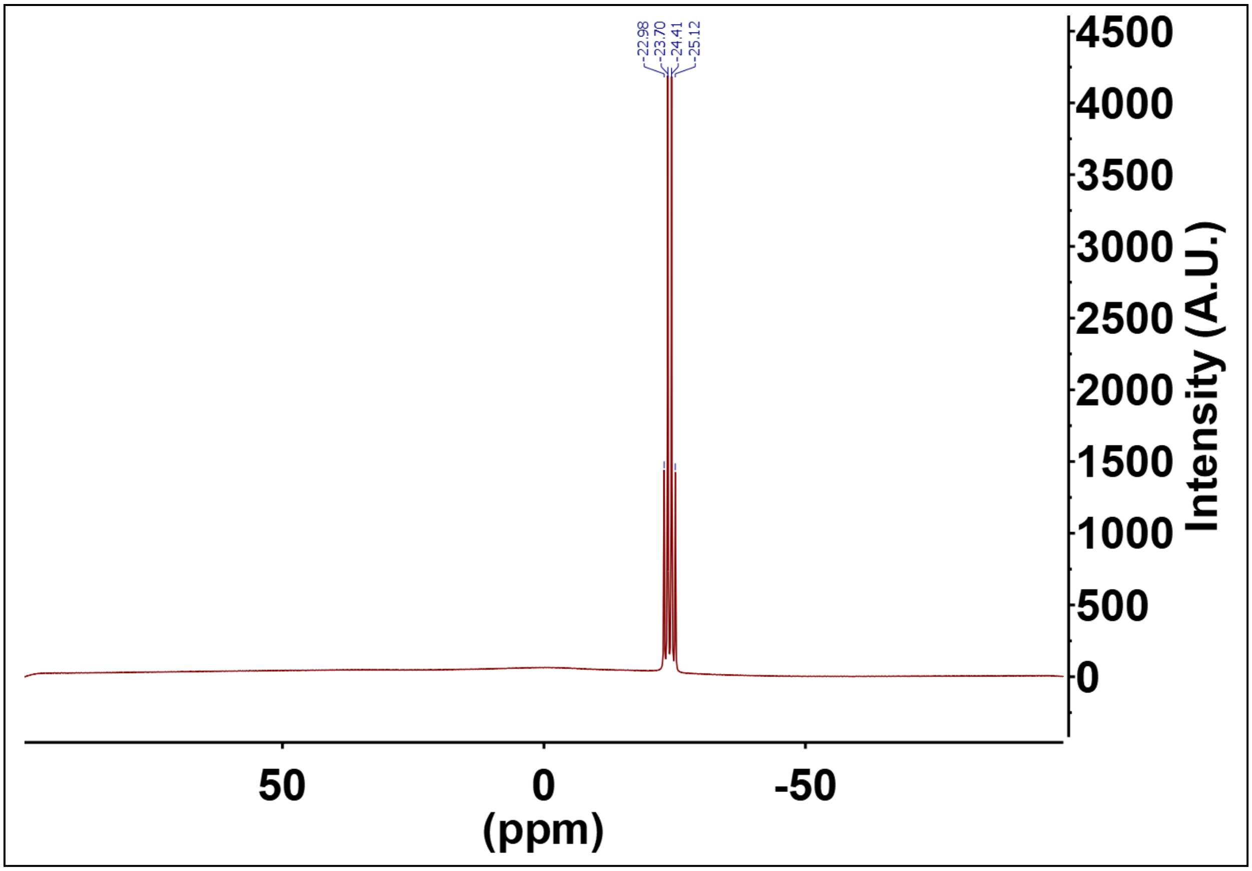

Figure S13. ^{11}B NMR spectrum of ammonia borane (after purification) synthesized using commercially available struvite from the reaction stopped at 24 hours. Percent yield is 21%.

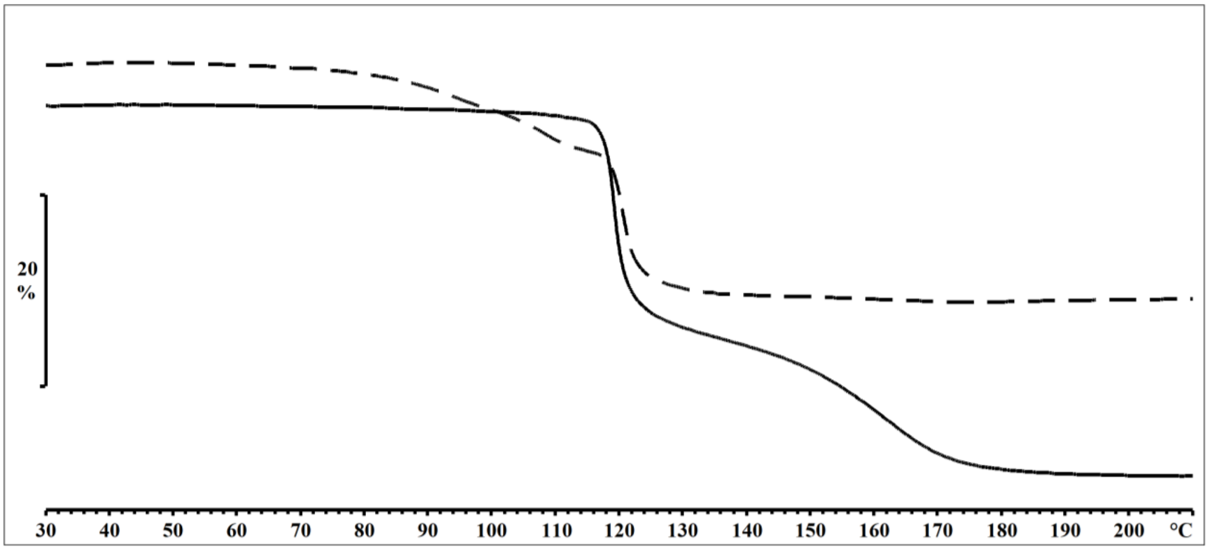

Figure S14. Thermogravimetric analysis of ammonia borane synthesized using commercially available struvite from the reaction stopped at 24 hours. A dash line represents crude product and a solid line represents product after purification.
